# Microfluidics-enabled phenotyping of a whole population of *C. elegans* worms over their embryonic and post-embryonic development at single-organism resolution

**DOI:** 10.1038/s41378-018-0003-8

**Published:** 2018-05-07

**Authors:** Maria Cristina Letizia, Matteo Cornaglia, Raphaël Trouillon, Vincenzo Sorrentino, Laurent Mouchiroud, Maroun S. Bou Sleiman, Johan Auwerx, Martin A. M. Gijs

**Affiliations:** 1 Ecole Polytechnique Fédérale de Lausanne, Laboratory of Microsystems, Lausanne, Switzerland; 2 Ecole Polytechnique Fédérale de Lausanne, Laboratory of Integrative Systems Physiology, Lausanne, Switzerland

## Abstract

The organism *Caenorhabditis elegans* is a performant model system for studying human biological processes and diseases, but until now all phenome data are produced as population-averaged read-outs. Monitoring of individual responses to drug treatments would however be more informative. Here, a new strategy to track different phenotypic traits of individual *C. elegans* nematodes throughout their full life-cycle—i.e., embryonic and post-embryonic development, until adulthood onset, differently from life-span—is presented. In an automated fashion, single worms were synchronized, isolated, and cultured from egg to adulthood in a microfluidic device, where their identity was preserved during their whole development. Several phenotypes were monitored and quantified for each animal, resulting in high-content phenome data. Specifically, the method was validated by analyzing the response of *C. elegans* to doxycycline, an antibiotic fairly well-known to prolong the development and activate mitochondrial stress-response pathways in different species. Interestingly, the obtained extensive single-worm phenome not only confirmed the dramatic doxycycline effect on the worm developmental delay, but more importantly revealed subtle yet severe treatment-dependent phenotypes that are representative of minority subgroups and would have otherwise stayed hidden in an averaged dataset. Such heterogeneous response started during the embryonic development, which makes essential having a dedicated chip that allows including this early developmental stage in the drug assay. Our approach would therefore allow elucidating pharmaceutical or therapeutic responses that so far were still being overlooked.

## Personalized medicine: Microfluidics-assisted individual drug response measurement

A microfluidic device containing millimeter-sized, single animal chambers allows researchers to detect previously-unseen individual drug responses in *Caenorhabditis elegans* worms. Martin Gijs and his research group from EPFL, Switzerland, developed their system wherein individual *C. elegans* organisms can be cultured throughout their entire life-cycle, while preserving their individual identity. The group then validated their system by testing the animals’ responses to a drug (doxycycline) known to induce certain biological changes. Alongside the standard, expected effects experienced by the majority of the animals, the researchers were also able to detect significant individual responses, starting in early-stage worm development, that would have otherwise been overlooked in a standard population-based study. The group’s system facilitates automation, offers clear advantages over some similar, previously reported techniques, and could provide substantial benefits to the human drug discovery process.

## Introduction

Heterogeneity in response to drugs or treatments can impede the quality of care, thus spurring the development of individualized therapeutic strategies^1,2^. The growing interest in personalized medicine is moving pharmacological research towards the preliminary identification of sub-groups of patients who will benefit from a specific treatment, from those for whom the treatment will not be effective or even deleterious. Such a priori knowledge would allow tailoring the therapy to the characteristics of each individual, rather than to a population-averaged behavior. To capture the individual variability, longitudinal experiments are needed, where large datasets are collected while maintaining the possibility to observe the response of a single entity. Big data approaches can be applied to identify different sub-groups, correlate them with markers and phenotypes and therefore provide new patterns predicting the response to the drug or treatment.

Given the cost and high attrition rate of human clinical trials, animal models are still widely used in early stages of drug development to elucidate the therapeutic mechanisms and to extrapolate the drug response to humans. The nematode *Caenorhabditis elegans* is a popular animal model for the analysis of complex traits, such as metabolism and aging, at the molecular, cellular, organ, and organismal levels^3^. However, the vast majority of the current biological knowledge, for any model, is largely based on population measurements, hiding potentially relevant individual behaviors. In the case of *C. elegans*, the majority of the experimental repertoire is based on analyzing and phenotyping large groups of individuals, hindering the crucial individualized spatiotemporal mapping. Microfluidics has been instrumental in enabling single-worm analysis with robust, high-throughput, and reproducible screenings^4–7^. However, it was not exploited for systematic phenotypic profiling covering embryonic and post-embryonic development of whole single organisms. Authors reported phenotypic platforms, in which cohorts of adult worms were confined in a culture chamber and phenotypic features, such as body size, motility and fluorescence expression, were monitored^8^. However, the lack of single-animal resolution impeded longitudinal phenotypic studies. Single worm isolation was performed either by using droplets, agarose compartments or by confining animals in tapered channels and traps. Animals were encapsulated in droplets starting from the fourth larval (L4) stage^9^ and even from the first larval (L1) stage^10^ and monitored over time, up to worm’s death^11,12^. A preliminary study was reported, where eggs were isolated in droplets and new-born worms were kept alive for 4 days in the droplet^13^. However, when performing droplet worm-isolation, delivery of fresh feeding solutions and chemicals is still challenging. Similarly, larvae were manually isolated in agarose micro-compartments and then cultured^14,15^. This approach still requires long manual preparatory steps and lacks the possibility of active fluidic exchange, crucial for automation purposes. A few screenings on single animals have been shown, where L4 or adult worms were temporarily confined in customized channels, while short behavioral and anatomical features were observed and measured^16–18^. Also longitudinal observations on single worms were performed. To this purpose, previously synchronized populations of adult worms or larvae were synchronized off-chip and injected into the device, isolated in tapered channels or by multilayer pressure valves, before being confined in a culture chamber. Different phenotypes were monitored, such as worm size, motility, progeny number, mating and development time^16,19–21^. However, in these cases worm synchronization was performed either by manual and time-consuming synchronizations off-chip or by using aggressive bleaching steps that could compromise the integrity of the organism. Moreover, typically, these analysis were performed starting from the L4 stage of the worms, thus preventing monitoring phenotypic variations occurring during worm embryonic and post-embryonic development. Studies were reported, where hydrodynamic or dielectrophoretic forces were exploited for long term trapping and imaging of zebrafish or nematode *Panagrolaimus davidi* embryos on chip^22–24^. Recently, a protocol to automatically collect and isolate embryo directly from egg laying on-chip, avoiding bleaching or manual procedures, and monitor embryonic development was shown^25^. However, a comprehensive quantitative and dynamical analysis of phenotypes in single *C. elegans* from embryo to adulthood was not performed yet. It is clear that a platform allowing single-worm based multi-phenotypic measures upon a specific treatment for the entire life-cycle would pave the way for extensive high-content, individualized analysis and possibly targeted follow-up treatments.

In this context, this article describes an innovative chip-based strategy for the precise analysis of the heterogeneous response to pharmacological treatments—and the resulting identification of heterogeneous biomarkers or phenotypes—amongst a controlled *C. elegans* population over the embryonic and larval development. In this endeavor, a microfluidic platform was designed to automatically isolate naturally-synchronized embryos upon egg laying and culture them into single-worm chambers, where the identity of each organism was preserved during its life-cycle. Several phenotypes were monitored for each worm, resulting in high-content phenome data for each experimental condition. Once all the individual phenotypes were extracted, one could match the different experimental conditions with phenotypic signatures. Multivariate methods were employed to navigate the datasets and identify only the most important phenotypes for each experimental condition. We validated our method by analyzing the response of *C. elegans* to doxycycline, an antibiotic known for extending the life-span of different experimental models and for activating the mitochondrial unfolded protein response (UPR^mt^), a stress-response that is conserved across species^26–28^. UPR^mt^ induction during larval development in *C. elegans* leads to reduced mitochondrial function^26,29^, as well as developmental delay and decreased brood size^26,29,30^. However, the relevance of these phenotypes to the lifespan extension, as well as their relative variations after UPR^mt^ induction, is still unclear. The single-worm-based analysis method was therefore employed to examine these treatment-dependent phenotypes. Interestingly, the population-averaged response to doxycycline matched previous results confirming the doxycycline-dependent extension of the worms‘ life-cycle duration. However, more importantly our single-worm-based phenotyping analysis additionally revealed subtle but significant responses characteristic of sub-groups of animals, observed during both the embryonic and the larval development, and that would have not be spotted using a different device and phenotyping approach.

## Materials and methods

### Fabrication of the microfluidic chips

**Fig. 1:**
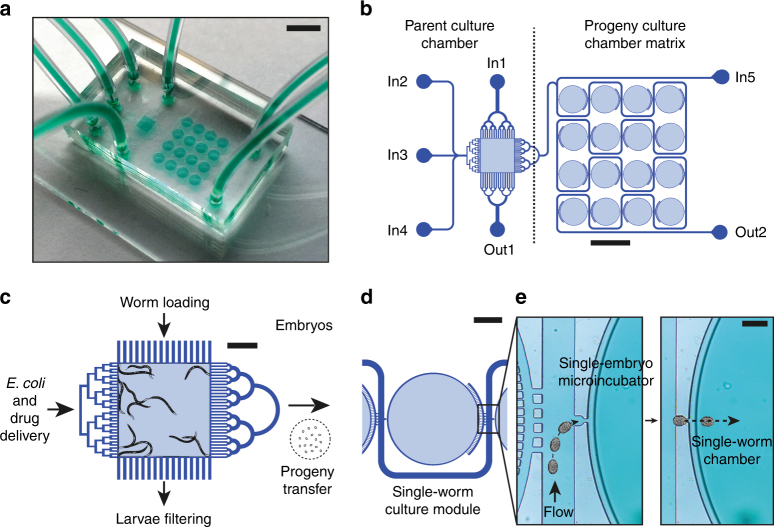
Automated platform for isolation and monitoring of single *C. elegans* organisms during full life-cycle. **a** Picture of the microfluidic device. Scale bar = 5 mm. **b** Schematic of the microfluidic device, showing the parent culture chamber and the progeny culture chamber matrix. Scale bar = 2 mm. **c** Zoom on the parent culture chamber including typical drawings of young adult *C. elegans*. Scale bar = 500 μm. **d** Schematic representation of a module of the progeny culture chamber matrix. Scale bar = 500 μm. **e** Sketch of the egg hydrodynamic trapping in a single-embryo micro-incubator and its transfer in the adjacent single-worm chamber. Scale bar = 100 μm

Microfluidic chips were prepared by soft lithography^31^ using 2-layer SU-8 on a silicon mold. First, the mold was fabricated by traditional photolithography. After fabricating a 40 μm-thick SU-8 pattern on a silicon 4-inch wafer, the second 90 μm-thick SU-8 layer was patterned on top of the first. Secondly, such a mold was used for polydimethylsiloxane (PDMS) casting. We poured a liquid PDMS mixture with a weight ratio base:curing agent of 10:1, waited for degassing and cured at 100 °C for 1 h. Thirdly, after de-molding, we diced each PDMS 13 mm × 23 mm chip, and punched holes into the PDMS to form inlets and outlets, and bonded each to a 26 mm × 76 mm glass slide, upon air-plasma surface activation. Finally, tubings were plugged in the PDMS holes. A picture of the final glass-PDMS microfluidic device is given in Fig. 1a.

### Platform control and image acquisition

The microfluidic chip was integrated onto an inverted microscope (Axio Observer, Zeiss) equipped with a digital camera (Hamamatsu, Japan) and two illumination systems: (i) a precisExcite High-Power LED Illumination system (Visitron, Puchheim, Germany) for brightfield imaging and (ii) a Lambda DG4 illumination system (Sutter instruments, Novato, CA, USA) for fluorescence imaging. The microscope was equipped with a motorized xyz-stage that had an ASI piezo controller for z-displacement (Visitron, Puchheim, Germany) and the automated imaging process was controlled using VisiView Premier Image acquisition software (Visitron, Puchheim, Germany). To start the automated imaging process, we initialized the position of either each embryo-incubator or each single-worm chamber as single point of the xyz-stage scanning. We also programmed the acquisition system so that brightfield (exposure time = 2 ms) and fluorescent (exposure time = 150 ms) pictures were recorded every 7 min on each position. A Zeiss 20 × NA 0.22 objective was used for imaging of single-embryos in the micro-incubator, while a Zeiss 4 × NA 0.075 objective was used for imaging of each single-worm chamber. A single picture was taken to capture each worm at a time-point, as the field of view of the objective was large enough to accommodate the whole single-worm chamber. An immobilization method was not needed, as the mobility of the worms was low. Indeed, the presence of optimized amounts of food along the duration of the whole experiment limited food-seeking behavior and motion. To capture sharp fluorescent pictures, the exposure time was finely tuned to avoid extensive animal movement within the exposure time window. The fluorescent picture was captured 150 ms (fluorescent exposure time) after the brightfield picture. Afterwards, the motorized stage moved to the following single-worm chamber and waited 3 s for stabilization, before brightfield and fluorescent pictures were shot. In this way, we could image the full array in about 50 s. Considering the typical timescale of the evolution of the development of the worms (~10 h for embryonal development and 2–5 days for larval development), this imaging routine is not expected to introduce a development mismatch between the different worms. The microfluidic operations were controlled using Nemesys syringe pumps (Cetoni, Korbussen, Germany). Given the relatively long time scale of each experiment, the syringe dedicated to nutrient *Escherichia coli* (*E. coli*) delivery was provided with a custom-made stirring system in order to prevent bacteria from sedimenting at the bottom of the syringe. We placed a magnetic rod (1 mm × 1 mm × 2 mm) (Supermagnete, Uster, Switzerland) inside the syringe. A rigid tube with small magnet rods blocked inside and fixed to the axis of a DC motor 6/9 V (Arduino, Ivrea, Italy) was positioned adjacent to the syringe. The rotation of the motor axis and its rods induced agitation in the magnet inside the syringe. The rotation of the motor axis was controlled by a microcontroller (Arduino, Ivrea, Italy) and it was synchronized to the syringe control, so that *E. coli* was stirred before being injected into the microfluidic system.

### Microfluidic platform operation

A schematic of the microfluidic device is reported in Fig. 1b. The microfluidic chip and tubing were first filled with a solution of Pluronic F-127 injected from inlet In2 and incubated overnight, in order to prevent *E. coli* sticking and clogging inside the microfluidic channels^32^. A mixed population of worms was suspended in 200 μL of S medium and sucked up in a tube connected to the device. Afterwards, with a flow of 500 nL/s, worms were injected in the microfluidic chip from inlet In1 in the parent culture chamber, where they were automatically synchronized. Outlet Out1 was provided with tailored filters, designed in such a way that only young adult (YA) and adult worms were retained in the chamber, while younger worms were washed out by the flow. The number of worms retained in the chamber was controlled by the concentration of YA and adults (typically from 5 to 10 worms) in the 200 μL solution. After worm synchronization, outlet Out1 was closed, while Out2 was opened. Worms were then exposed to a constant flow of fresh S medium at 15 nL/s from inlet In3, and a constant flow of 20 nL/s of *E. coli* bacteria—containing treatment when needed—from inlet In4. With this flow program, we not only provided medium and nutrients to the parental population until worms were ready for egg laying, but also automatically and simultaneously delivered freshly-laid embryos towards the progeny culture chamber matrix. In the doxycycline experiment, embryos were treated with a solution of doxycycline in *E. coli* provided from inlet In5. Once the full array of single-embryo micro-incubator was filled and the embryos reached the twitching-onset stage, we simultaneously transferred all the embryos from each incubator to the adjacent single-worm chamber, by applying a flow of S medium at 20 μL/s for 2 s from inlet In3, taking advantage of PDMS elasticity. After the embryo transfer, the parental generation was removed from the chip. To do so, outlet Out1 was opened and a flow of 20 μL/s was applied from inlet In1 until all the adult worms were washed out. Afterwards, we closed outlet Out1 as well as inlets In1, In2, In3, and In4. From this step on, only inlet In5 and outlet Out2 were used. A flow profile adapted to larval age was developed, in order to provide enough nutrients to the progeny generation (See Supplementary Fig. S1). During the embryonic twitching-hatching stage and during the larval 1 (L1) stage, we delivered a repetitive loop flow profile of 5 nL/s for 10 min and 20 nL/s for 15 s, in order to ensure that the newborn larvae could not escape through the single-embryo micro-incubator, as the incubator aperture dimension matched the L1 size. Afterwards, a loop flow profile of 10 nL/s for 15 min, 150 nL/s for 15 s and 0 nL/s for 15 min was used, in order to refresh *E. coli* bacteria in the chambers, to avoid stressing the worms with continuous hydrodynamic forces. The experiments were performed at 25 °C.

### Chemicals and materials

Four-inch 550 μm thick Si wafers, 5-inch chromium-glass masks, and de-ionized water (DIW) were obtained from the Center of Microtechnology and Nanotechnology at EPFL. GM 1070 SU-8 negative photoresist was purchased from Gersteltec (Pully, Switzerland). PDMS Sylgard 184 was acquired from Dow Corning (Wiesbaden, Germany). One milliliter polycarbonate syringes were purchased from BD (Franklin Lakes, NJ USA). Tygon tube with 0.51 mm inner and 1.52 mm outer diameters was bought from Fisher Scientific (Wohlen, Switzerland). Pluronic F-127 was purchased from Sigma-Aldrich (Buchs, Switzerland). S medium buffer was obtained by adding 1 L S Basal, 10 mL 1 M potassium citrate pH 6, 10 mL trace metals solution, 3 mL 1 M CaCl, 3 mL 1 M MgSO. Pluronic F-127 solution was prepared by diluting 0.4% (weight/volume) Pluronic F-127 in DIW. Doxycycline (Sigma), was dissolved in water to a stock concentration of 6 mg/mL.

### Image processing

Image processing was performed with ImageJ software (https://imagej.nih.gov/ij/, RRID = SCR_003070) and Matlab R 2015b software (MathWorks, U.S.A, RRID = SCR_001622), on each stack of brightfield and fluorescent worm images. Brightfield pictures were processed in order to spot bean stage onset, twitching onset, hatching, molting times and appearance of protruded vulva for each individual. The number of surviving embryos was assessed by counting the number of hatching eggs. For each larval stage, length and diameter (or width, as the worms are assumed cylindrical) were calculated and averaged over five brightfield pictures at the center of the larval stage for each individual. In order to assess fertility, we looked for the presence of internal embryos from self-fertile reproduction after vulva protrusion. To extract fluorescent signal-to-background (SBR) ratio, the region of interest (ROI) corresponding to the worm area was first segmented in the whole stack. We performed background subtraction and applied a median filter in order to remove salt-and-pepper noise. SBR was then calculated as SBR = (I_ROI_-I_BG_)/I_BG_ for each picture, with I_ROI_ average intensity of the signal in the ROI and I_BG_ the average intensity of the background. In order to remove noise and artifacts from the obtained SBR, a moving average filter (length of moving window = 10 frames) was applied on the stack.

### Experimental model and subject details

*C. elegans* strains were cultured at 20 °C on nematode growth media (NGM) 90 mm Petri dishes seeded with *E. coli* strain OP50 (RRID = WB-STRAIN:OP50). The strain used in this study was the SJ4100 (zcIs13[hsp-6p::GFP] RRID = WB-STRAIN:SJ4100), provided by the Caenorhabditis Genetics Center (University of Minnesota). For studies in untreated conditions, *hsp-6p::gfp* worms were plated on regular NGM plates at the L4 stage, and collected about 12 h later at the stage of YA for injection in the system. For the doxycycline treatment, a concentration of 30 μg/mL was chosen to trigger UPR^mt^ in *hsp-6p::gfp* worms, as previously described on nematode growth medium (NGM)^29,33^. Adult gravid worms were placed on control plates and removed after 6 h after egg laying. F1 YA worms were then collected for introduction in the microfluidics system, where the treatment with the antibiotic (30 μg/mL) was initiated after egg laying. Worms were suspended in S medium solution prior to each microfluidic experiment.

### *E. coli* culture

HT115 *E. coli* bacteria (RRID = WB-STRAIN:HT115) were grown in Luria Broth (LB) with 100 μg/mL ampicillin and 12.5 μg/mL tetracycline overnight in a thermal shaker at 37 °C. The following day, 50 µL of the confluent bacterial cultures were used to inoculate freshly prepared LB medium containing only ampicillin, so to avoid the presence of tetracycline during the worm experiments, which has been shown to also affect mitochondrial function, similarly to doxycycline^33^. The new cultures were grown until reaching an optical density between 0.6 and 0.8, and 90 μL were used for seeding the experimental plates.

### Quantification and statistical analysis

The on-chip analysis provided a multitude of phenotypic variables which precluded the identification of major phenotypic changes in response to the experimental conditions. As a consequence, we used a hierarchical cluster analysis and principal component analysis (PCA) (see SI) to reduce the dimensionality of the datasets and emphasize the driving force that generated the raw data.

The input experimental variables were the durations of the embryonal stages of development, the durations of the five stages of larval development, the length at the L1 stage and length increments at the other four stages of larval development, and the fluorescent SBR at L1 stage and the SBR increments at the other four stages of larval development. Increments, rather than absolute measurements, were used here to minimize the risk of unwanted correlations between parameters which are expected to increase as the worm develops (for instance the length), which may hide smaller variations between the treatments. Each dataset was normalized and centered to the average of each parameter for the negative control, and a hierarchical cluster analysis was run using the built-in algorithm in Matlab R 2015b (MathWorks, USA, RRID = SCR_001622). Data points were organized in clusters ordered according to the unweighted average Euclidean distance. Statistical analysis was performed using GraphPad Prism (San Diego, CA, USA, RRID = SCR_002798). Graphs are expressed as mean ± SD overlaid with a cloud of points describing the actual dataset, unless otherwise indicated in the figure legends. For each treatment, the datasets were independently obtained from two chips, each featuring 16 worm chambers. The sample number (N) indicates the number of independent viable biological samples for which parameters were extracted in each dataset. Statistical significance was determined with a two-tailed Mann–Whitney test. *p*-values were graphically reported as *p ≤ 0.05, **p ≤ 0.01. Untreated *hsp-6p::gfp* worms are used as controls. Statistical tests were performed only to compare the recorded values of a given phenotypic parameter, at a specific developmental stage, amongst different treatments/groups of worms.

## Results

### Single-worm high-content phenotypic platform

A microfluidic chip for the phenotypical analysis and biomarker identification at single-worm level throughout its life-cycle was designed and fabricated in polydimethylsiloxane (PDMS) and glass (Fig. 1a). First, a non-synchronized population of *C. elegans* was loaded in the parent culture chamber (Fig. 1b, c). In order to synchronize the population in an automated way, dedicated filters were fitted to outlet Out1 to retain only young adult (YA) worms in the chamber and wash younger worms out. Afterwards, those YA worms were fed with *E. coli* bacteria until egg production. Once laid, embryos were transported by the flow to the progeny culture chamber matrix (Fig. 1b, d), consisting of an array of 16 single-worm chambers, each fitted with a trap for single-embryo capture (Fig. 1e). Through hydrodynamic trapping the embryos occupied consecutive traps, forming a naturally-synchronized population^25^. More details about the design of the hydrodynamic trapping system are provided in the SI. Using this approach, the traditional aggressive bleaching step and the time consuming manual synchronization procedures were avoided^34^. The single-embryo traps were presented and discussed in a previous study published by our group^25^. The device used in the current work pushes further this concept by connecting a worm-culture chamber to each single-embryo trap (Fig. 1d, e). With this approach, the new-born larva remains isolated and can be cultured until adulthood onset, allowing for the first time the on chip monitoring of embryonic and post-embryonic development. In fact, after capture, each embryo was simultaneously injected from the micro-incubator to the adjacent single-worm chamber by increasing the flow rate, exploiting PDMS elasticity (Fig. 1e). Brightfield and fluorescent pictures were automatically taken at a fixed rate, first of each single-embryo micro-incubator and then of each single-worm chamber, from egg laying until adulthood onset. This guaranteed sufficient temporal resolution to describe transitions between the different development stages.

**Fig. 2:**
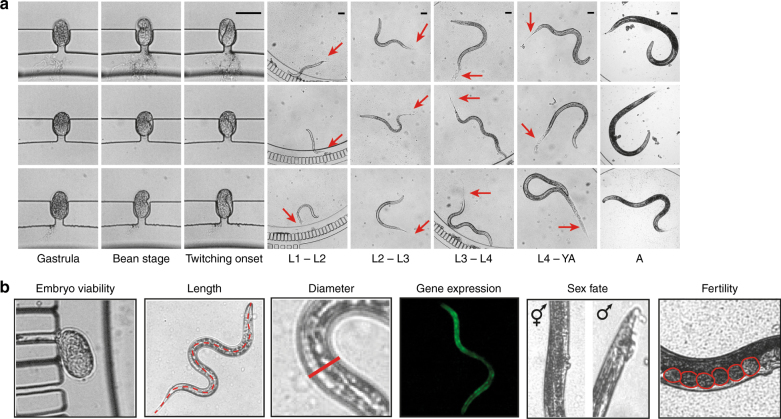
Single-worm based phenotypes extraction. **a** Illustration of the parallel time-lapse imaging, here shown only for three worms, enabled on the worm array within the progeny culture chamber matrix during the full life-cycle. Arrows point at the molts seen as detached from the worm body. Scale bar = 50 μm. **b** Illustration of the quantified phenotypic markers, namely embryo viability or hatching rate, worm length and diameter at the vulva at the stages L1, L2, L3, L4 and YA, the fluorescence expression of the gene of interest at the stages L1, L2, L3, L4 and YA, the sex fate and the percentage of fertile adult worms

We identified the milestones characterizing embryonic and larval development^35,36^, detailed in Fig. 2a. *C. elegans* embryos, laid upon beginning of gastrulation, were immediately trapped in the embryo-incubator array. Bean stage and twitching onset were observed in the single-embryo incubator, while egg hatching was detected in the single-worm chamber. The duration of each larval stage could be unambiguously evaluated by measuring the time interval between consecutive moltings, which identify the actual biological transitions amongst the different larval stages (L1 to L4), up to the YA stage. The molting events are the perfect indicators of the larval development status and spotting all the four moltings of each animal represents a unique feature of our single-worm culture chip. Adulthood onset was defined by the vulva protrusion, which normally coincides with the appearance of first self-fertilized embryos inside the worms, and which determined the end of the life-cycle and therefore our experiment. Other important phenotypes were extracted for each larval stage of development, for each animal (Fig. 2b): the hatching rate, the average body length and diameter at the vulva, and the expression level of a gene of interest via fluorescent read-out. Moreover, the sex fate (hermaphrodite/male) and the fertility rate were recorded as well.

### Phenotypic biomarker analysis of a control strain

**Fig. 3:**
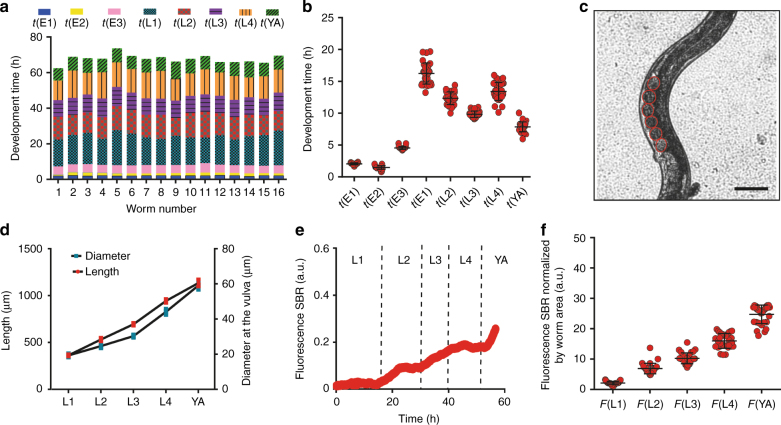
Phenotypic analysis of the *hsp-6p::gfp* strain at single-organism resolution. **a** Duration of the main embryonic and larval developmental phases, observed for a matrix of 16 worms. **b** Durations of embryonic and larval developmental stages. **c** Brightfield picture of an adult worm. Scale bar = 50 μm. **d** Average length and diameter at each larval stage. Graph points are expressed as mean ± SD. **e** Quantification of *hsp-6p::gfp* expression as signal-to-background ratio (SBR) for the full life-cycle. **f** Quantification of *hsp-6p::gfp* expression as SBR normalized by worm size at each larval stage. *N* = 31

High-content phenotypic data on *hsp-6p::gfp* worms, a nematode UPR^mt^ reporter strain^37^ that can be considered, except for the fluorescent expression, equivalent to N2 wildtype strain, was obtained by extracting biologically-informative phenotypic markers from the images. The durations of the developmental stages along the life-cycle—gastrula-bean stage t(E1), bean stage-twitching t(E2), twitching-hatching t(E3), larval stages t(L1), t(L2), t(L3), t(L4) and t(YA) stage—were systematically quantified for individual worms (16 per array, Fig. 3a) and averaged over the array (Fig. 3b). In the absence of treatment, the duration of these stages matched those reported for worms cultured on agar plates at the same temperature^38^. Moreover, 97% of the trapped embryos developed and 100% of them became fertile hermaphrodite adults, as internal eggs were observed (Fig. 3c). The average worm lengths, corresponding to the skeleton (later abbreviated as L(L1), L(L2), L(L3), L(L4) and L(YA)), and diameters, at each larval stage were also obtained (Fig. 3d). The basal induction of the *hsp-6p::gfp* was quantified as a reporter of the UPR^mt^ activation by processing the fluorescent images to extract the signal-to-background ratio (SBR). The linear SBR increase over time (Fig. 3e) suggests that the mitochondrial stress pathway is weakly activated in basal conditions^29,37^. The fluorescent signal per worm area at each larval stage (F(L1), F(L2), F(L3), F(L4), F(YA)) was also computed (Fig. 3f).

### Phenotypic analysis upon doxycycline treatment

**Fig. 4:**
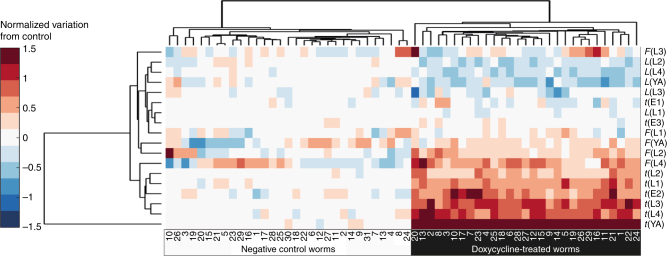
**Hierarchical cluster analysis of phenotypes of the control group and the treated group**

The platform was used to phenotypically characterize the effect of doxycycline. Cluster analysis (Fig. 4) and principal component analysis (PCA) (Supplementary Fig. S2 and Supplementary Table 1) were first run on the dense datasets to identify features of interest, and provide an overall phenotypic footprint of the effect of doxycycline on the treated worm population. This antibiotic induced severe deviations from control, as treated animals showed longer development time t(L3), t(L4) and t(YA), confirming the doxycycline role in prolonging the animal development and extending its lifespan.

**Fig. 5:**
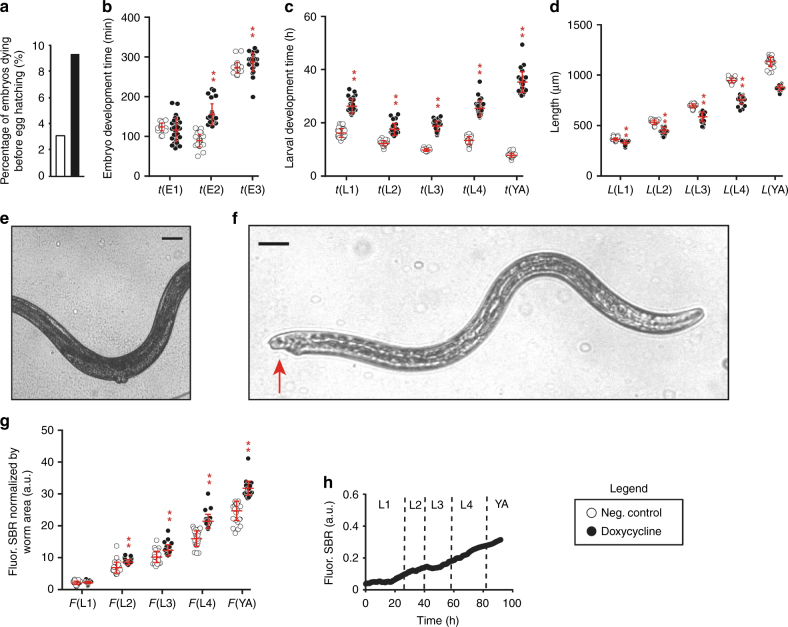
Doxycycline-induced mitochondrial stress response monitored during the *C. elegans* life-cycle. **a**–**d** Comparison between control and treated worms of **a** the percentage of embryos dying before egg hatching, **b** the durations of embryonic developmental stages, **c** the durations of larval developmental stages and **d** the lengths at each larval stage. **e** Brightfield picture of the abdomen of an adult treated worm showing no presence of eggs in the body. Scale bar = 50 μm. **f** Brightfield picture of one of the adult treated worms that expressed a male sex. The arrow points at the characteristic male tail. Scale bar = 50 μm. **g** Comparison of SBR normalized by worm size at each larval stage between control and treated worms. **h** Quantification of *hsp-6p::gfp* expression as SBR for the full life-cycle. *N* = 31 for control; *N* = 29 for the doxycycline treatment. Untreated *hsp-6p::gfp* worms are used as controls

Interestingly, the unexpected permeability of the eggshell to doxycycline induced two different responses on the embryo population. The doxycycline treatment proved to be lethal for 9.4% of the embryos, that died before hatching (Fig. 5a). This response is in line with the effect of other mitochondrial stressors^39,40^. On the other hand, the embryo development of the surviving eggs was severely delayed (Fig. 5b), especially during the bean-twitching phase t(E2).This effect was also observed in a previous study using a genetic mutation to induce mitochondrial stress^25^. As predicted by cluster analysis, the impact of doxycycline was even more severe on the larval development time (Fig. 5c) and size (Fig. 5d and Supplementary Fig. S3), which featured a 1-fold or higher increase over the negative control, especially for the L4 and the YA stages. This observation agrees with previous reports showing a similar delay in larval development when the electron transport chain function is compromised genetically^41^. Indeed, during the L3–L4 stages, mitochondria undergo a period of dramatic proliferation^42^, making it the critical time-window, during which mitochondrial perturbations can have large impact on *C. elegans* larval development^41^. Moreover, doxycycline not only impairs gonad development and causes infertility (Fig. 5e), but also pushes 8% of the individuals towards male development (Fig. 5f). These findings suggest that doxycycline exerts profound effects on developmental processes relying on mitochondrial support, favouring a shift towards male progeny formation, similar to what is observed with other sources of stress^43^, but, interestingly, only in a subgroup of the population. Finally, the tracking of *hsp-6p::gfp* expression (Fig. 5g, h), showed an overall increase starting at the L2 larval stage, which confirms the induction of mitochondrial stress by doxycycline, followed by the UPR^mt^.

## Discussion

Quantifying and elucidating the patterns linking markers to heterogeneous responses amongst treated individuals is a paramount prerequisite in precision medicine research. However, this variability in phenome data read-out is often lost owing to the lack of specific strategies to tackle this huge challenge. Consequently, we described here a platform that allows culturing and phenotyping of individual worms from a *C. elegans* population from egg laying until adulthood. Thanks to hydrodynamic trapping, freshly-laid single embryos were automatically isolated and—for the first time—kept in separate chambers until adulthood. The single-worm resolution of our chip allowed the longitudinal monitoring and tracking of multiple phenotypes over the life-cycle of individual organisms. Moreover, worms showed a physiological development consistent with the one in solid medium at the same temperature, thus setting a new standard in terms of *C. elegans* culture and feeding in liquid environment. Therefore, with respect to previous studies on *C. elegans* culture^14,25^ and its use in drug testing^18,25^, we showed a comprehensive microfluidic platform enabling the unprecedented *C. elegans* culture over its full life-cycle at the single-worm level while longitudinally monitoring multiple phenotypic markers upon a variety of treatments. In contrast with solid medium approaches, the platform allowed testing a pharmacological treatment by simply injecting the compound of interest into the microfluidic chip from a dedicated inlet, while ensuring perfect control and reproducibility of all the environmental parameters. Conjugating longitudinal assays over the full development span with individualized observations in a precisely controlled chemical environment is a clear advancement of the state of the art and a substantial improvement of the previously proposed analytical microfluidic platforms for *C. elegans*. This phenotyping strategy paves the way for the identification of appropriate disease biomarkers that would shape the worm response to the treatment of interest, which, would complement ideally similar studies performed on other species (reviewed in ref. 44). To compare the high-content phenotypic signature of worm populations under different conditions, we used single-worm based hierarchical clustering analysis and principal component analysis. Big data strategies facilitate the identification of sub-groups and patterns buried in the phenome data. The method also evaluates quantitatively the differences among individuals, and provided the phenotypic footprint of each treatment. Envisioning a personalized medicine in future, the characterization of such intra-population treatment-dependent variability will allow both inferring the effectiveness of a treatment in a population’s subsets and identifying personalized follow-up treatments.

The platform and analysis method were used to identify appropriate biomarkers and investigate the phenotypic variability associated with the doxycycline treatment. The classic phenotypic hallmarks of mitochondrial stress were observed, such as delayed development, reduced worm size, reduced fertility and induction of the UPR^mt^ biomarker *hsp-6p::gfp*. However, the setup also revealed a surprising treatment-dependent intra-population variability that could otherwise not be observed in experiments run on plates or with different microfluidic culture platforms. Indeed, at the beginning of the life-cycle, doxycycline was lethal to a small group of embryos that could not complete the development, while it prolonged the embryonal development of the others. Moreover, during the gonad development, doxycycline affected the sex fate of a subgroup of embryos that eventually developed as males and not as the usual hermaphrodites. Finally, the fact that the heterogeneous response was observed during both the embryonic and the larval development, confirms the actual need for a microfluidic culture device that allows performing the drug treatment, and monitor its effect, throughout the full animal’s life-cycle.

## Conclusions

We presented a versatile microfluidic platform, configured with a modular high-content phenotypic analysis that could include in the future longitudinal analysis over the full life-cycle of phenotypes as diverse as motility, pharyngeal pumping and expression of different reporter genes. This microfluidic-based phenotyping strategy, combined with advanced analysis of the clinical and molecular phenomes, could be used to identify markers related to treatments whose effects are poorly characterized. Moreover, we envision that this platform could be widely employed for identifying single worms as responders or non-responders to a treatment based on their phenotypic footprint. It might also facilitate phenotypic analysis upon RNAi screenings where intrinsically a strong variability is to be expected^3^. Translation of these findings to humans could open breakthroughs in early phases of drug development and medicine through follow-up treatments tailored to the characteristics of the patient.

## Electronic supplementary material

Supplemental material

Doxycycline data set and principal component analysis
