# Supplementary material for: Microfluidics-enabled phenotyping of a whole population of C. elegans worms over their embryonic and post-embryonic development at single-organism resolution
Source: Microsyst Nanoeng. 2018 May 7;4:6. doi: 10.1038/s41378-018-0003-8 (PMC6220190; doi:10.1038/s41378-018-0003-8)
Supplement: Supplementary file 1 — Supplemental material [file 41378_2018_3_MOESM1_ESM.pdf]

SUPPLEMENTARY FILE

Microfluidics-enabled phenotyping of a whole population of *C. elegans* worms over their embryonic and post-embryonic development at single-organism resolution

Maria Cristina Letizia, Matteo Cornaglia, Raphaël Trouillon, Vincenzo Sorrentino, Laurent Mouchiroud, Maroun S. Bou Sleiman, Johan Auwerx, Martin A.M. Gijs

FEEDING FLOW PROFILE

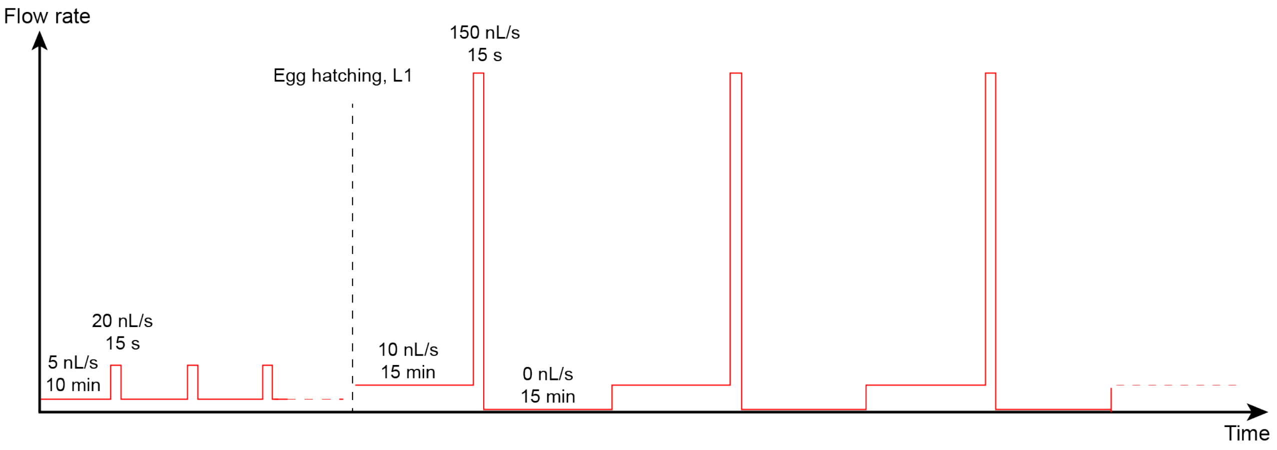

Supplementary Figure S1. Bacterial flow profile used to perfuse the chamber over the full experiment. Different flow profiles, adapted to the worm's stage of development, were chosen for embryonal and larval development.

PRINCIPAL COMPONENT ANALYSIS (PCA)

As data (Table S1) are spread over a wide range of units and scales, we normalized the data to the mean of each parameter (obtained over all the worms for a specific condition, *i.e.* control and treatment) before performing PCA. This allowed for the direct comparison of the different parameters while still preserving the variability of the data, in regard to the average, hence allowing for the extraction of the discriminant

markers. Additionally, the algorithm used here centers the dataset by default. A PCA procedure was run using the built-in algorithm in Matlab R 2015b (MathWorks, USA). The components were then ranked by decreasing order of explained variance (or eigen-value). The data was then plotted using this new base, and the components revealing the separation of specific subgroups were kept for the analysis. For each retained component the contribution of the initial parameters was computed by normalizing the coordinates of the component to the L1 norm of the component (i.e. the sum of all the absolute values of the coordinate for this component). Only the parameters whose contribution was at least 10% of the component were considered. Finally, by considering the projection of the raw data on the relevant components (i.e. by considering the scores) and the parameters contributing to this component, the discriminant parameters for specific subgroups were identified. Using this approach, the contribution of each phenotypic observation could be evaluated, and the input variables with the highest information content isolated. Results are given in Supplementary Figure S2.

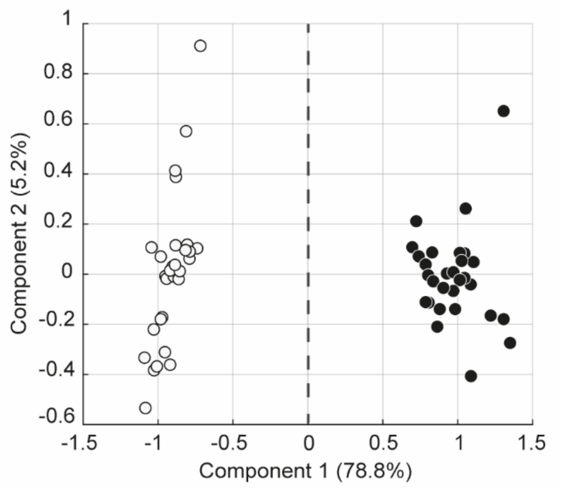

Supplementary Figure S2. Principle component analysis (PCA) of phenotypes of control and treated groups. Single-worm data are arranged according to the first and the second principle component of worms' phenotypes.

EFFECT OF DOXYCYCLINE

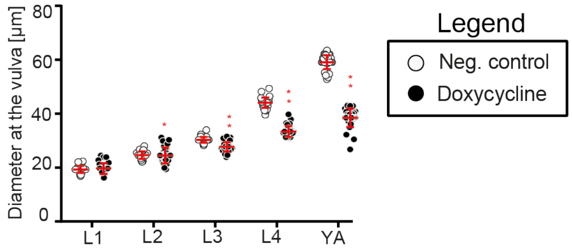

Supplementary Figure S3. These supplemental data complete the datasets shown in Figure 5. Comparison of diameters at each larval stage between negative control and doxycycline-treated worms. N= 31 for control; N= 29 for the doxycycline treatment. Untreated *hsp-6p::gfp* worms are used as controls.

ENGINEERING THE HYDRODYNAMIC TRAPPING SYSTEM

The efficiency of our embryo trapping mechanism was predicted by calculating the ratio between the volumetric flow rates through each chamber and its bypass "serpentine" channel, respectively. In very first approximation, this ratio can be theoretically estimated by considering the pressure drop between inlet and outlet of each chamber along the two parallel fluidic paths and imposing equal pressure difference along them, assuming Poiseuille flow conditions [1]. This calculation, however, ignores losses due to channel bends, widening/narrowing, etc., which are indeed relevant in our design because of its composite geometry. In particular, spreading flow profiles at the boundary of pinched and broadened segments are completely neglected under this assumptions, which make therefore this calculation a very rough estimate. Nevertheless, we decided to employ these calculations as a tool for a first approximated dimensioning of our chips, whose architecture was then refined through the results of a systematic experimental characterization. Assuming Poiseuille flow conditions, the ratio between the flow rate through a chamber and the one through its bypass channel can be calculated as the inverse of the ratio between the hydraulic resistances of the two fluidic paths (Supplementary Figure 4a). For a straight

channel with rectangular cross-sectional shape, the hydraulic resistance R can be calculated using Poiseuille Equation $R = \frac{12\mu L}{1-0.63\frac{h}{w}}\frac{1}{h^3 w}$.

In our case, the resistance of the bypass channel, R_{bypass}, can be approximated using Poiseuille Equation, while the fluidic path across the culture chamber is calculated as a series of 3 main resistances (Supplementary Figure 4a): (i) $R_{incubator}$, including the resistances of a single-embryo trap and its drain connection to the chamber; (ii) the chamber resistance, $R_{chamber}$ and (iii) R_{outlet}, made up of two interconnected series of parallel resistances.

We estimated the trapping efficiency for 6 similar versions of our device and fabricated them to experimentally determine the hydraulic resistance ratio which maximizes the capture efficiency in our geometry. For these tests, 30 µm polymeric beads were employed as trapping objects and define the capture efficiency as the percentage of traps containing a single bead over the total number of available trapping sites, for a constant amount of beads injected into the microfluidic device. From our experimental analysis, we conclude that a ratio R_{bypass} / ($R_{incubator}$ + $R_{chamber}$ + R_{outlet}) of ~2.6 maximizes the system trapping efficiency (Supplementary Figure 4b).

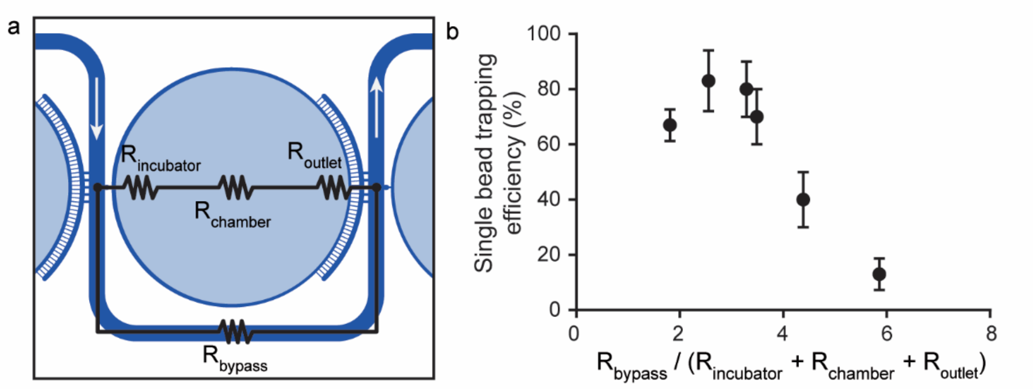

Supplementary Figure S4. Engineering of the hydrodynamic trapping system. (a) Schematic representation of the design of the hydrodynamic embryo trapping system, with indication of the hydraulic resistances associated to the different parts of the microfluidic geometry. (b) Experimental characterization of the trapping efficiency of the system for 6 similar microfluidic designs (characterized

by different hydraulic resistances in their main constitutive parts). The “single bead trapping efficiency” is measured as the percentage of traps capturing a single 30 μm bead over the total number of available trapping sites.

REFERENCES

1. Tan, W.-H. & Takeuchi, S. A trap-and-release integrated microfluidic system for dynamic microarray applications. *Proc. Natl. Acad. Sci.* **104,** 1146–1151 (2007).
