# Supplementary material for: Microfluidics-enabled phenotyping of a whole population of C. elegans worms over their embryonic and post-embryonic development at single-organism resolution
Source: Microsyst Nanoeng. 2018 May 7;4:6. doi: 10.1038/s41378-018-0003-8 (PMC6220190; doi:10.1038/s41378-018-0003-8)
Supplement: Supplementary file 2 — Doxycycline data set and principal component analysis [file 41378_2018_3_MOESM2_ESM.pdf]

Table S1

			Phenotypic markers																			PCA scores	
			Embryo development [min]			Larval development time [h]					Larval length [µm]					Fluorescent signal (normalized by area) [a.u.]						
		Worm #	Stage 1	Stage 2	Stage 3	L1	L2	L3	L4	YA	L1	L2	L3	L4	YA	L1	L2	L3	L4	YA	PC 1	PC 2
Experimental data	Control	w1	118	50	272	15.112	12.73	9.341	11.019	6.987	360.464	524.011	695.435	930.091	1129.876	2.041	7.024	10.154	19.811	27.324	-1.043	0.108
		w2	135	110	265	16.527	11.113	9.648	15.304	7.803	362.845	537.878	698.018	939.918	1120.488	2.201	7.187	10.008	14.493	24.126	-0.861	-0.017
		w3	115	120	278	17.601	11.316	10.212	12.133	8.283	353.024	485.021	649.425	916.552	1027.33	1.869	8.664	13.002	13.851	19.149	-0.790	0.087
		w4	130	95	266	14.584	12.123	10.563	14.7	7.702	402.396	558.251	719.488	955.403	1022.506	2.43	4.803	8.124	11.481	25.084	-0.973	-0.175
		w5	115	91	271	19.674	13.485	10.851	13.766	7.933	339.721	543.014	712.542	975.611	1184.356	2.614	6.002	9.601	19.124	22.176	-0.825	0.095
		w6	119	84	269	17.812	13.311	9.802	13.159	7.388	362.421	529.113	696.904	951.154	1135.521	2.087	7.042	10.058	14.168	27.451	-0.914	0.018
		w7	125	75	284	15.517	12.716	9.216	14.545	7.652	367.445	562.145	698.992	961.459	1206.157	1.039	5.524	8.003	11.621	21.185	-1.082	-0.535
		w8	130	75	278	14.747	13.474	9.121	15.511	7.816	348.661	529.41	663.767	933.46	1161.952	3.239	6.059	12.29	16.803	26.002	-0.892	0.415
		w9	121	92	269	16.397	10.019	9.812	12.158	9.816	361.913	539.095	690.761	942.097	1125.135	2.507	7.174	10.022	14.641	27.131	-0.880	0.117
		w10	128	85	277	15.401	13.621	9.691	12.614	8.257	359.425	488.143	698.865	920.587	1165.901	2.913	13.701	15.476	16.942	27.704	-0.719	0.913
		w11	122	108	315	14.412	14.411	9.853	14.019	7.534	363.446	525.251	702.877	951.705	1127.156	2.091	7.254	10.021	14.146	26.361	-0.861	0.018
		w12	140	85	271	16.501	12.251	9.941	13.059	5.966	362.128	544.201	696.081	936.578	1130.094	2.207	7.458	10.324	14.178	26.784	-0.983	0.070
		w13	130	90	257	14.451	12.481	9.758	13.416	7.816	388.136	545.389	699.008	969.967	1129.084	1.299	6.252	9.201	11.455	22.141	-1.027	-0.385
		w14	100	100	272	16.452	11.937	9.902	11.05	9.084	360.651	528.011	690.054	931.056	1134.843	2.011	7.085	10.004	14.462	25.212	-0.887	-0.013
		w15	130	80	253	17.151	11.082	9.354	12.661	7.535	361.098	538.994	718.188	993.714	1162.086	1.946	5.104	7.211	13.842	18.921	-1.096	-0.334
		w16	121	79	275	19.5	10.85	10.504	12.95	7.786	353.667	562.695	709.667	939.989	1077.284	1.09	5.801	9.706	18.22	26.355	-0.918	-0.361
		w17	119	59	271	15.512	11.37	9.808	15.971	7.612	365.411	533.552	695.765	946.475	1119.15	2.022	6.015	10.163	19.241	27.045	-0.945	-0.009
		w18	136	108	258	16.254	12.456	10.955	10.132	6.922	362.775	541.355	682.018	936.085	1142.634	2.007	7.301	11.067	16.254	26.032	-0.907	0.031
		w19	114	121	275	17.616	11.219	10.295	13.133	8.278	354.271	487.192	650.926	926.094	1043.27	1.806	8.605	13.125	17.651	19.805	-0.740	0.104
		w20	129	94	264	13.245	12.139	10.5	14.712	8.475	405.586	569.698	723.985	969.452	1098.766	2.403	4.861	8.325	15.481	17.687	-0.981	-0.179
		w21	114	91	268	19.601	13.605	10.746	14.761	7.987	341.705	541.004	722.429	965.615	1186.569	2.691	6.046	9.614	18.825	22.072	-0.810	0.115
		w22	121	89	272	18.28	12.3	9.204	13.558	7.85	362.334	527.622	690.068	928.845	1128.094	2.032	7.003	11.076	15.187	27.045	-0.882	0.036
		w23	124	76	279	15.516	12.616	9.217	14.584	7.753	354.458	552.541	688.982	980.863	1201.675	1.352	5.591	8.085	17.697	21.86	-1.013	-0.372
		w24	131	77	285	14.3	13.416	9.514	15.506	7.814	349.689	532.856	685.985	958.865	1175.276	3.109	6.042	12.298	18.803	26.095	-0.877	0.389
		w25	119	89	270	14.417	12.001	9.441	12.236	7.732	360.094	541.654	679.971	942.244	1128.121	2.081	6.058	10.005	18.158	27.041	-0.954	-0.008
		w26	131	84	280	15.4	13.76	9.66	12.623	8.183	354.387	486.424	689.462	900.528	1168.662	2.939	10.003	12.476	15.941	27.74	-0.814	0.570
		w27	118	111	314	16.35	11.901	9.412	13.532	7.022	358.149	530.005	685.987	951.297	1124.648	2.017	7.751	10.217	14.125	27.706	-0.879	0.037
		w28	141	84	265	16.504	12.179	9.471	14.684	7.582	365.09	528.175	703.047	920.085	1118.054	2.101	7.074	10.285	18.285	26.817	-0.886	0.034
		w29	129	91	260	14.405	12.403	9.808	13.499	7.831	391.614	549.254	705.255	949.547	1144.875	1.295	6.201	9.213	19.254	22.288	-0.958	-0.314
		w30	101	99	274	16.817	12.379	9.922	12.254	10.047	362.035	534.542	656.781	920.451	1124.452	2.166	7.048	10.111	17.002	27.009	-0.788	0.062
		w31	129	80	255	17.15	12.089	9.333	12.645	7.588	351.505	525.989	721.902	997.857	1168.529	1.947	5.4	8.012	14.421	26.91	-1.025	-0.222
	Treated	w1	135	145	199	26.016	17.85	16.833	26.351	41.766	338.501	461.818	621.648	763.23	863.512	1.212	8.419	12.268	22.149	31.381	1.091	-0.407
		w2	133	133	294	25.4	16.466	21.987	30.693	32.783	350.973	492.154	580.133	752.981	822.192	2.363	9.686	10.942	21.806	29.555	0.970	0.006
		w3	182	161	292	24.51	15.175	19.135	27.121	35.816	294.996	448.155	579.213	684.018	838.357	2.297	9.435	12.124	19.037	30.148	0.974	-0.071
		w4	120	153	312	30.416	17.583	21.233	22.465	35.119	282.015	389.014	483.014	661.962	886.804	2.415	7.978	11.164	21.456	32.169	1.046	-0.018
		w5	79	144	297	32.666	15.433	19.959	24.726	37.011	343.219	487.753	565.033	744.029	898.683	3.288	7.87	12.412	20.348	29.397	1.055	0.258
		w6	82	156	283	27.034	17.6	16.818	25.941	34.533	352.87	424.646	626.874	754.485	890.104	2.116	8.472	12.138	22.479	32.475	0.929	0.003
		w7	115	202	297	23.683	15.233	18.366	23.933	30.454	348.051	492.321	589.963	756.343	905.502	2.022	7.927	12.191	20.692	32.126	0.796	-0.117
		w8	184	138	314	26.133	16.994	15.416	26.251	33.839	328.791	443.476	627.417	780.392	915.782	1.745	10.544	11.916	20.398	30.435	0.781	-0.112
		w9	95	125	285	24.666	16.216	15.993	23.689	33.366	336.212	476.417	549.032	804.414	844.145	2.51	8.289	11.763	21.297	30.602	0.693	0.106
		w10	107	219	248	29.517	16.8	20.379	23.458	31.038	347.418	471.874	619.72	756.811	852.085	2.354	9.975	12.103	21.516	33.874	1.044	0.083
		w11	154	158	273	30.219	15.416	19.491	22.112	35.424	326.599	491.353	649.618	783.252	812.444	2.061	9.653	14.889	25.553	33.352	1.019	0.086
		w12	131	141	298	26.563	19.483	21.718	27.183	35.355	349.147	448.905	597.787	800.504	900.867	2.841	8.015	10.796	20.856	31.854	1.031	0.052
		w13	145	129	315	28.747	21.7	18.583	35.466	30.216	318.642	389.963	509.451	729.371	889.572	2.269	8.152	10.992	24.987	30.959	1.016	-0.026
		w14	89	137	299	24.521	18.083	16.917	25.083	31.551	302.874	492.475	537.143	647.918	851.862	2.425	8.417	11.012	19.975	30.624	0.741	0.070
		w15	146	148	304	24.853	15.633	19.366	29.166	35.893	336.951	443.451	546.425	761.712	873.917	2.271	8.108	10.945	20.784	31.983	0.981	-0.136
		w16	70	157	289	22.983	15.05	17.894	27.185	32.274	328.082	448.874	631.148	809.829	902.582	1.475	8.414	15.785	21.742	32.927	0.835	-0.030
		w17	112	198	294	24.195	16.451	18.314	26.566	31.966	348.749	447.802	600.514	798.904	904.913	2.005	7.855	10.981	20.341	28.677	0.870	-0.212
		w18	126	139	322	24.383	16.213	17.619	22.983	34.654	347.014	416.354	589.563	749.718	899.445	2.438	8.147	12.014	20.876	31.761	0.791	0.039
		w19	84	124	298	26.366	17.383	16.286	23.696	32.268	336.151	465.122	627.456	748.679	901.437	2.436	8.541	13.417	21.475	33.517	0.727	0.209
		w20	75	151	281	23.457	22.75	18.327	27.066	37.066	328.145	491.041	483.089	694.788	871.476	2.427	10.864	18.468	30.115	41.157	1.310	0.647
		w21	104	216	259	31.733	23.216	19.483	23.583	39.588	302.053	443.547	616.944	754.863	863.371	1.878	7.741	11.209	20.324	32.679	1.349	-0.277
		w22	140	141	271	28.875	18.272	22.497	26.366	40.833	348.579	448.747	600.685	785.114	900.485	2.245	8.632	10.874	20.479	31.525	1.219	-0.165
		w23	105	208	262	23.8	21.475	19.252	22.052	35.233	297.704	384.451	489.754	720.402	879.177	2.366	8.525	12.437	21.238	31.658	1.105	0.048
		w24	98	136	281	23.916	17.733	20.533	22.416	49.357	336.074	443.701	614.248	794.016	863.337	2.248	7.897	11.274	19.746	31.729	1.308	-0.178
		w25	77	154	287	25.474	17.578	20.066	28.659	37.089	328.745	482.745	627.582	758.356	884.243	2.215	8.182	12.314	20.136	31.522	1.081	-0.043
		w26	124	147	295	25.284	16.179	17.971	24.654	33.155	336.721	459.768	607.457	775.142	870.387	2.094	8.491	13.375	20.534	31.481	0.807	-0.007
		w27	119	151	290	24.826	17.164	20.798	22.547	35.479	334.175	461.545	605.175	780.145	872.146	2.195	8.397	10.746	19.467	28.944	0.886	-0.133
		w28	123	150	294	26.947	18.818	16.954	23.678	36.476	338.713	457.189	629.179	779.146	868.546	2.139	8.601	11.758	21.485	31.658	0.929	-0.058
		w29	121	146	293	25.176	15.678	17.894	23.165	34.537	336.472	460.315	608.144	784.063	870.998	2.294	8.143	14.567	20.745	32.569	0.829	0.086

		PC 1 (78.8% of variance)		PC 2 (5.2% of variance)	
		Coordinates	Contributions	Coordinates	Contributions
**Embryo development [min]**	**Stage 1**	-0.035	0.011	-0.140	0.049
	**Stage 2**	0.292	0.092	-0.098	0.034
	**Stage 3**	0.024	0.007	0.061	0.021
**Larval development time [h]**	**L1**	0.253	0.080	-0.050	0.018
	**L2**	0.191	0.060	0.066	0.023
	**L3**	**0.337**	**0.106**	-0.069	0.024
	**L4**	**0.326**	**0.103**	-0.075	0.026
	**YA**	**0.688**	**0.217**	-0.172	0.060
**Larval length [µm]**	**L1**	-0.049	0.015	-0.034	0.012
	**L2**	-0.085	0.027	-0.036	0.013
	**L3**	-0.089	0.028	-0.054	0.019
	**L4**	-0.117	0.037	-0.042	0.015
	**YA**	-0.133	0.042	0.027	0.010
**Fluorescent signal (normalized by area) [a.u.]**	**L1**	0.034	0.011	**0.655**	**0.230**
	**L2**	0.125	0.039	**0.461**	**0.162**
	**L3**	0.103	0.032	**0.460**	**0.162**
	**L4**	0.157	0.050	0.177	0.062
	**YA**	0.135	0.042	0.169	0.059

Table legend

Stage 1	egg laying - bean stage
Stage 2	bean stage - twitching onset
Stage 3	twitching onset - hatching
